# Association between nitrogen dioxide and incident breast cancer in Alberta’s tomorrow project

**DOI:** 10.1038/s41598-025-04373-x

**Published:** 2025-05-30

**Authors:** Mohadeseh Ahmadi, Rachel A Murphy, Maryam Darvishian, Trevor J. B. Dummer

**Affiliations:** 1 School of Population and Public Health, Faculty of Medicine, University of British Columbia, Vancouver, Canada; 2 Cancer Control Research, BC Cancer, Vancouver, Canada

**Keywords:** Built environment, Post-menopausal, Breast cancer, NO_2_, TRAP, Breast cancer, Risk factors, Environmental impact

## Abstract

Breast cancer is a significant global health concern, with millions of cases diagnosed annually. Risk factors for breast cancer include obesity, physical inactivity, poor diet, and alcohol use. Urbanization introduces exposures such as traffic-related air pollution (TRAP), which may impact breast cancer risk. To assess the impact of TRAP, measured in this study using nitrogen dioxide (NO_2_), on the risk of post-menopausal breast cancer we conducted a secondary analysis of baseline and follow-up data from the Alberta Tomorrow Project (ATP) cohort, linked to geographic data at the postal code level from the Canadian Urban Environmental Health Research Consortium (CANUE). Cox proportional hazard regression models were built to assess the association between breast cancer risk and NO_2_. Direct acyclic graphs (DAG) were used to identify and select relevant confounders for adjustment. The study included 15,536 post-menopausal women and 523 incident breast cancer cases over 12.6 years of follow-up. The hazard ratio (HR) for the initial model, which included all potential confounders (age, education, number of births, fruits and vegetables consumption, income, physical activity, BMI, and smoking) was 1.01 per 10-ppb increase in NO_2_. The adjusted HR for a 10-ppb increase in NO_2_ measured at baseline (the year participants completed the health and behaviours survey) was 1.10 (95% CI = 0.90, 1.34). Our study found no significant association between NO_2_ exposure and post-menopausal breast cancer risk. The variation in reported hazard ratios (HRs) — ranging from 1.01 to 1.10 — reflects different models adjusting for different variables. Our findings suggest a risk magnitude similar to other studies, yet the lack of significant association may be due to sample size limitations and lower NO_2_ levels in Alberta compared to other regions globally.

**Supplementary Information:**

The online version contains supplementary material available at 10.1038/s41598-025-04373-x.

## Introduction

Breast cancer is a significant disease among women, with approximately 2.3 million cases diagnosed globally in 2020, resulting in 685,000 deaths^1^. In Canada, about 84 women receive a breast cancer diagnosis every day^2^. Established risk factors for post-menopausal breast cancer, which account for around two thirds of all cases, include obesity, physical activity (PA), dietary intake, smoking, family history of breast cancer, and alcohol^3–8^.

A large proportion of breast cancer cases occur in highly developed countries, often associated with urbanization and economic growth^9^. Currently, about half of the global population reside in urban areas, a figure projected to increase to more than 90% by 2100^10^. Living in urban areas offers many benefits, including better healthcare access, but it may also expose individuals to elevated environmental pollution levels^11^, primarily from air pollution and stationary-source fuel combustion^12^. Traffic-related air pollution (TRAP), a mixture of various compounds, such as carbon dioxide (CO_2_), nitrogen dioxide (NO_2_), sulfur dioxide (SO_2_), ozone (O_3_), and particulate matter (PM)^13^, has been identified as a Group 1 carcinogen by the International Agency for Research on Cancer (IARC)^14^. Recent studies suggest a potential causal link between these compounds, specifically NO_2_, and female breast cancer, especially in post-menopausal women^15–18^.

Although there has been extensive research on the association between NO_2_ and other chronic conditions, such as lung cancer and cardiovascular diseases, its association with breast cancer is less well studied. While some studies reported positive and significant associations between breast cancer and NO_2_ exposure, others have found non-significant associations. In a large US-based cohort study, no overall increase in breast cancer risk per interquartile range difference (5.8 parts per billion - ppb) in NO_2_ exposure was observed (HR = 1.02, 95% CI: 0.97, 1.07)^18^. In the European ESCAPE cohort study, which combined data from 15 prospective European cohorts, there was a suggestive positive association between exposure to most ambient air pollutants and post-menopausal breast cancer^19^. However, the association between ambient NO_2_ and breast cancer did not reach statistical significance, with a HR of 1.02 per 10 µg/m^3^^19^. Two large cohort studies, in the Catalonia region of Spain and in Canada, reported a modest non-significant increase of breast cancer incidence with greater NO_2_ exposure. In the Canadian National Breast Screening Study, while no association was observed between breast cancer risk and NO_2_ in the post-menopausal cohorts, in pre-menopausal women, using 50- and 52-years to identify pre- and post-menopausal women, a 9.7 ppb increase in NO_2_ exposure (per interquartile range increase) was linked to rate ratios (RR) of 1.13 and 1.17^16^. The Catalonia study, on the other hand, reported a HR of 1.05 (95% CI, 1.02, 1.08) per interquartile range (27.6 µg/m^3^) increase in NO_2_ exposure in post-menopausal women^20^.

In line with these mixed findings, significant uncertainty remains regarding a causal association between air pollution, particularly NO_2_, and post-menopausal breast cancer. Further research is needed to fully elucidate the mechanisms linking NO_2_ to breast cancer.

The objective of the present study was to assess the association between NO_2_ exposure and post-menopausal breast cancer risk in Alberta’s Tomorrow Project (ATP), a longitudinal cohort study that enrolled participants aged 35 to 69 residing in the province of Alberta, Canada. Given the age range of participants in ATP the majority of breast cancer cases were post-menopausal, and the analysis was restricted to the post-menopausal women given the distinct differences between pre- and post-menopausal breast cancer^3^.

## Methods

### Study population

The ATP is a longitudinal cohort study that enrolled participants through two phases of recruitment. Phase I, conducted between 2000 and 2008, employed a two-stage telephone random digit dialing (RDD) approach to recruit participants. In this phase, the RDD was mapped to Alberta Regional Health Authorities (RHA) to select households with eligible residents. One or two residents from each household were then invited for participation in the study. Phase II, spanning from 2009 to 2015, enrolled participants through two methods: first, by extending invitations to individuals already involved in Phase I to join Phase II, and second, via volunteer sampling. The recruitment process for Phase II did not employ RHA-based RDD. Between 2000 and 2015, ATP recruited 55,000 men and women between the ages of 35 and 69 years, from towns and cities across the province, with the goal of long-term follow-up over 50 years. Baseline information for individuals recruited during Phase I was collected through the Health and Life Questionnaire (HLQ), Canadian Dietary History Questionnaire (CDHQ), and Physical Activity Questionnaire (PAQ). For participants recruited during Phase II, the same baseline data were collected using an Updated Health and Life Questionnaire (UHLQ) and Physical Activity and Nutrition Survey (PANS). Starting in January 2011, the UHLQ and PANS were replaced by the Canadian Partnership of Tomorrow Project (CanPath) CORE Questionnaire. Consequently, participants who joined the study in 2011 or later exclusively completed the CORE Questionnaire. Our study focused on post-menopausal women. Women were considered post-menopausal either through their response to the ATP questionnaire (options included: yes – natural menopause, yes – other reasons, no, don’t know) or their age at enrollment. In the ATP questionnaire, menopausal status was defined as whether an individual’s menstrual period stopped for one year and did not restart. Further, in cases where the participant answered “no” and were over the age of 55, the age cut-off of 60 was used. In cases where the participant answered “don’t know” or did not answer the question, then the age cut-off of 55 years was used to ascertain menopausal status^21^. This study received ethical approval from the University of British Columbia Behavioral Research Ethics Board. We confirm that all methods were performed in accordance with the relevant guidelines and regulations.

### Outcome classification

In the present study, cases were defined as post-menopausal individuals who experienced incident, in situ, or invasive breast cancer, and were censored at the year of diagnosis. Incident cases were identified through record linkage to the Alberta Cancer Registry, and data on breast cancer diagnosis, such as tumor staging and other pathological characteristics, were not extracted for this study. Post-menopausal participants who had been diagnosed with breast cancer prior to their enrollment in the study were excluded. Non-cases were post-menopausal women who did not develop breast cancer during the follow-up period (follow-up end date: June 30, 2022), with outcomes including mortality, loss to follow-up, or reaching the end of the follow-up period.

### Exposure assessment for NO_2_

The analysis focused on NO_2_ as a marker or proxy of TRAP. We accessed this information through the Canadian Urban Environmental Health Research Consortium (CANUE) dataset, which compiles and generates standardized environmental data at the dissemination area level related to different built environment characteristics. Following data compilation, CANUE facilitates the integration of the data into existing Canadian cohort studies and administrative health databases, including the ATP database. CANUE data was linked to ATP based on participants’ postal code of residence at enrolment. The initial land-use regression (LUR) annual average concentration at postal code in 2006, which represented annual average concentration at baseline, were provided for all participants in parts per billion (ppb).

### Analytic population

The ATP recruited both men and women; however, the study’s dataset included only women, as men were excluded based on the applied exclusion criteria before the data was provided. This aligns with the study’s focus on post-menopausal breast cancer in women. Individuals were considered eligible for inclusion if they were either post-menopausal at the time of enrollment or transitioned into the post-menopausal stage during the follow-up period. These menopausal status transitions were identified through the questionnaires that participants completed or based on their age at baseline. In addition, participants with follow-up time of less than one year were excluded from analysis. With this exclusion criteria, in some cases, incident breast cancers diagnosed within one year of enrolment were also eliminated from the study. In instances where participants had two breast cancer diagnoses at different time points, only first diagnosis was retained.

### Statistical analysis

In this study, multivariable Cox proportional-hazards regression models were used. We built a model for NO_2_ measured at baseline (the year participant completed the UHLQ or CORE survey) – and estimated HRs and 95% confidence intervals for the association between incident breast cancer and 10 ppb change in NO_2_. Follow-up time was used as the time scale for the Cox model, calculated as the difference between the year of enrollment and the year of diagnosis for cases, or between the year of enrollment and the end of follow-up in 2022. Schoenfeld’s tests assessed Cox proportional-hazards assumptions, while a 5% change-in-estimate threshold guided the selection of potential confounders for final models, alongside a directed acyclic graph (DAG) to identify a minimally sufficient adjustment set.

The covariate adjustment set included body mass index (BMI) (underweight - BMI less than 18.5 kg/m^2, normal weight - BMI between 18.5 and 24.9 kg/m^2, overweight - BMI between 25 and 29.9 kg/m^2, and obese - BMI greater than or equal to 30 kg/m^2), physical activity (met or did not meet World Health Organization PA recommendations), ethnicity (white or other), vegetable and fruit consumption (categories of 0, 1, 2, 3, 4, 5 or more), social and material deprivation (categorized into quintile of 1 – least deprived to 5 – most deprived), smoking (never smokers, daily smoker, and occasional smoker), income (less than 50 K, 50–100 K, 100–150 K, more than 150 K), alcohol consumption (never, less than once a month, once to 3 times a month, once per week, 2 to 5 times a week, and more than 6 times a week), education (category A - no formal education, elementary school, or high school; category B - included trade/technical or vocational school, a diploma from a community college, or a university certificate below bachelor’s level; category C comprised a bachelor’s degree or a graduate degree), contraceptive use (never or ever), birth (2 or less, 3–4, greater than 4), maternal breast cancer history (yes or no), and age (less than 44, 45–54, 55–64, more than 65). Social deprivation represents the deprivation of relationships within family, workplace, and community – the index is calculated based on the proportion of the population who are separated, divorced, or widowed; the proportion living alone; and the proportion who have relocated in the past five years^22^. Material deprivation represents the deprivation of goods and amenities – its index is based on average household income, unemployment rate, and the rate of high school education^22^.

First, we constructed the initial model (model I), which included all covariates. Next, based on a 5% cut-off for change-in-estimate procedure, variables including age, education, number of births, fruit and vegetable consumption, income, physical activity, BMI, and smoking met the criteria for inclusion (model II). However, guided by the DAG and existing literature (see supplementary Figure S1), only social deprivation, material deprivation, income, education, and age were classified as preliminary confounders. However, in model III, age, education, and social deprivation were retained. Social deprivation was included instead of income and material deprivation due to redundancy in the effects captured by these variables. We further constructed three models to assess the association between NO_2_ quartiles and postmenopausal breast cancer. Model I included all covariates. In the change-in-estimate model (model II), only age, number of births, and material deprivation model were retained. In the DAG-informed model (model III), the same confounders as previously identified (age, education, and social deprivation) were included, with social deprivation retained over material deprivation and income due to redundancy in their effects.

Physical activity had the highest missing value rate at 69%, while education and contraceptive use had the lowest at less than 1%. Most other variables had missing value rates below 15%, except for ethnicity, maternal history of breast cancer, and smoking, which all had rates around 30–40%. Notably, age was the only variable devoid of any missing values. To handle high missingness, missing data was categorized into a “not reported” category.

**Fig. 1:**
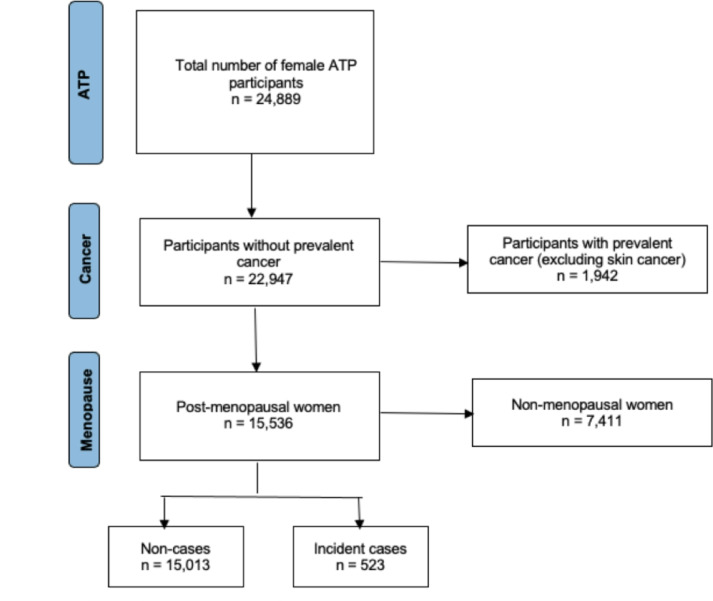
PRISMA diagram of study population. Abbreviations: ATP: Alberta’s Tomorrow Project.a.

There were 24,889 women who met all the specified criteria for this study. Among them, 12 participants had missing menopausal status data. From the 24,899 participants, 1,942 individuals had prevalent cancers at study baseline, excluding nonmelanoma skin cancer, which resulted in a study population of 22,947 after exclusion. An additional 7,411 participants who were premenopausal throughout the follow-up period were excluded from the analysis. This retained a final study population of 15,536 participants, see Fig. 1. During the follow-up period, there were 523 incident breast cancer diagnoses, accounting for 3.4% of the participants. The remaining 15,013 individuals did not receive a breast cancer diagnosis and were classified as non-cases. For the analysis of baseline NO_2_ concentration a further 485 observations (including 9 breast cancer cases) were excluded due to missing NO_2_ data. The proportional hazard assumption for all models was met. In this study, NO_2_ concentration measurements were utilized as a proxy for TRAP due to data availability.

## Results

### Descriptive statistics

The study population included 21% of individuals under the age of 44 who were post-menopausal, with 12% of cases in this age group identified as post-menopausal. In addition, 29% were aged 45–54, 34% aged 55–64, and 17% were over 65. Among the 17,001 participants who completed the CORE questionnaire, 94% identified as White. Participants were followed for an average of 12.6 years (SD = 4.2), with cases followed for 7.9 years on average and non-cases for 12.8 years. The mean age at baseline for the study population was 53.5 years, with a standard deviation of 9.6 years. Detailed descriptive statistics among cases and non-cases are presented in Table 1. Variables that were statistically significantly different in the two groups included age, ethnicity, maternal history of breast cancer, physical activity, and BMI.

**Table 1:** Baseline participant characteristics.

Variables		Overall		Cases		Non-cases		P
		N or mean	% or SD	N or mean	% or SD	N or mean	% or SD	
Number of participants		15,536	100%	523	3.40%	15,013	96.60%	
Follow-up time (years)		12.6	4.2	7.9	4.9	12.8	4.1	
Age at baseline		53.5	9.6	56.9	8.7	53.4	9.6	< 0.05
Alcohol consumption	Never	1127	7.7	39	7.8	1088	7.6	0.6
	Less than once per month	2964	20.1	107	21.4	2857	20.1	
	1–3 times per month	3652	24.8	114	22.8	3538	24.9	
	Once per week	1623	11	47	9.4	1576	11	
	2–5 times per week	4229	28.7	151	30.1	4078	28.7	
	More than 6 times per week	1132	7.7	43	8.5	1089	7.7	
	Unknown/Missing	809	5.2	22	4.2	787	5.2	
Birth	Two or less	4334	32.3	146	32.7	4188	32.3	0.19
	3–4	7893	58.8	261	58.4	7632	58.8	
	Greater than 4	1196	8.9	40	8.9	1156	8.9	
	Unknown/Missing	2113	13.6	76	14.5	2037	13.6	
Race	White	10,140	94.9	312	59.7	9828	94.9	0.002
	Other	540	5.1	12	2.3	528	5.1	
	Unknown/Missing	4856	31.3	199	38	4657	31	
Education	High school or below	3697	23.8	131	25.1	3566	23.8	0.2
	Diploma or trade	5565	35.8	198	37.9	5367	35.8	
	University-level education	6266	40.4	193	37	6073	40.4	
	Unknown/Missing	--	--	--	--	--	--	
Contraceptive use	Ever	13,825	89	467	89.3	13,358	89	0.87
	Never	1704	11	56	10.7	1648	11	
	Unknown/Missing	--	--	--	--	--	--	
Deprivation score - social	Q1 (least deprived)	2698	18.2	92	18.9	2606	18.2	0.25
	Q2	2809	19	97	19.9	2712	19	
	Q3	2956	20	95	19.5	2861	20	
	Q4	3337	22.6	115	23.6	3222	22.5	
	Q5 (most deprived)	2985	20.2	88	18.1	2897	20.3	
	Unknown/Missing	751	4.8	36	6.9	715	4.8	
Deprivation score – material	Q1(least deprived)	4321	29.2	133	27.3	4188	29.3	0.31
	Q2	3572	24.2	124	25.5	3448	24.1	
	Q3	2832	19.2	95	19.5	2737	19.1	
	Q4	2510	16.9	83	17	2427	17	
	Q5 (most deprived)	1550	10.5	52	10.7	1498	10.5	
	Unknown/Missing	751	4.8	36	6.9	715	4.8	
Fruit consumption, servings/day	Zero	578	3.7	21	4	557	3.7	0.6
	One	3137	20.3	95	18.2	3042	20.4	
	Two	5107	33	190	36.4	4917	32.9	
	Three	3606	23.3	121	23.2	3485	23.3	
	Four	1915	12.4	58	11.1	1857	12.4	
	Five or more	1119	7.3	37	7.1	1082	7.3	
	Unknown/Missing	--	--	--	--	--	--	
Vegetable consumption, servings/day	Zero	--	--	--	--	--	--	0.33
	One	1862	12.1	74	14.2	1788	12	
	Two	4210	27.2	128	24.6	4082	27.3	
	Three	3918	25.4	141	27.1	3777	25.3	
	Four	2842	18.4	95	18.2	2747	18.4	
	Five or more	2450	15.8	81	15.5	2369	15.9	
	Unknown/Missing	--	--	--	--	--	--	
Income	Less than 50,000	3046	21.2	123	25.5	2923	21.1	0.09
	$50,000-$100,000	4889	34.1	169	35	4720	34	
	$100,000-$150,000	3125	21.8	99	20.5	3026	21.8	
	Greater than $150,000	3290	22.9	92	19	3198	23.1	
	Unknown/Missing	1186	7.6	40	7.6	1146	7.6	
Maternal history of breast cancer	Yes	4023	41.1	171	46.7	3852	41	< 0.05
	No	5759	58.9	195	53.3	5564	59	
	Unknown/Missing	5754	37	157	30	5597	37.3	
Physical activity	Met	514	10.8	15	12.6	499	10.7	< 0.05
	Not Met	4263	89.2	104	87.4	4159	89.3	
	Unknown/Missing	10,759	69.3	404	77.2	10,355	69	
Smoking	Never	7674	87.2	279	89.1	7395	87.2	0.14
	Occasionally	--	--	--	--	--	--	
	Daily	870	9.9	30	9.6	840	9.9	
	Unknown/Missing	6738	43.4	210	40.2	6528	43.5	
BMI	Underweight	--	--	--	--	--	--	< 0.05
	Healthy	5969	41.6	168	36.2	5801	41.8	
	Overweight	4697	32.7	152	32.7	4545	32.7	
	Obese	3523	24.6	142	30.5	3381	24.4	
	Unknown/Missing	1188	7.6	58	11.1	1130	7.5	

Abbreviations: BMI: body mass index; p: p-value; PA: physical activity; MET: metabolic equivalent time; SD: standard deviation; For all variables, proportions were calculated excluding missing values from the denominator, and proportions of missing values were calculated using the total sample size as the denominator. --: participant count < 10 suppressed.

### Distribution of NO_2_ exposure estimated by LUR

The mean concentration of baseline NO_2_ was 10.1 ppb, with a standard deviation (SD) of 4.4. The highest and lowest NO_2_ concentrations were 34.8 ppb and 2.1 ppb, respectively (see supplementary Figure S2). The average baseline NO_2_ concentration for cases and non-cases were 10.3 ppb (SD = 4.4) and 10.1 ppb (SD = 4.4), respectively.

### NO_2_ exposure (continuous) at baseline and breast cancer

The HR for a 10-ppb increase in NO_2_ was 1.14 (95% CI = 0.90, 1.45) in model I. Further adjustments for breast cancer risk factors (model II) yielded an HR of 1.01 (95% CI = 0.82, 1.24) for a 10-ppb increase in NO_2_. In the final model the HR for a 10 ppb increases in NO_2_ was 1.10 (95% CI = 0.90, 1.34) (Table 2). This model had a concordance value of 0.62. Based on the Schoenfeld’s test, the proportional hazard assumption was met. The global p-value was 0.08, and the p-value for NO_2_ was 0.20. There was a total of 15,051 observations across all models, of which included 514 breast cancer cases.

**Table 2:** Association for breast cancer risk with baseline NO_2_ (continuous).

Regression model	HR	95% CI	*P*-value
Model I*	1.14	0.90–1.45	0.30
Model II**	1.01	0.82–1.24	0.90
Model III***	1.10	0.90–1.34	0.36

Abbreviations: NO_2_: nitrogen dioxide; HR: hazard ratio; CI: confidence interval; *Adjusted for all covariates; **Adjusted for age, education, number of births, fruit and vegetable consumption, income, physical activity, BMI, and smoking; ***Adjusted for education, social deprivation, and age.

### NO_2_ exposure (quartiles) at baseline and breast cancer

The HR for the second quartile (Q2) were slightly above 1 in all models (ranging from 1.12 to 1.16) with p-values and 95% CIs indicating statistical non-significance (Table 3). For the third quartile (Q3) there was a borderline significant association in model I (HR = 1.27, 95% CI: 0.97–1.66), which further strengthened in model II (HR = 1.29, 95% CI: 0.99–1.67), and became statistically significant in model III (HR = 1.31, 95% CI: 1.01–1.68). Compared to Q1, no association was observed for the highest quartile (Q4) of NO_2_, with HRs close to 1 across all models. The global p-value for NO_2_ was 0.07.

**Table 3:** Association for breast cancer risk with baseline NO_2_ (quartile).

NO_2_ Quartile	Model I* HR (95% CI), *p*-value	Model II** HR (95% CI), *p*-value	Model III*** HR (95% CI), *p*-value
Q1 (lowest)	1.00	1.00	1.00
Q2	1.12 (0.85–1.45), 0.41	1.14 (0.88–1.48), 0.31	1.16 (0.90–1.50), 0.25
Q3	1.27 (0.97–1.66), 0.08	1.29 (0.99–1.67), 0.06	1.31 (1.01–1.68), 0.04
Q4	0.96 (0.70–1.30), 0.79	0.94 (0.71–1.24), 0.65	0.99 (0.75–1.29), 0.91

Abbreviations: NO_2_: nitrogen dioxide; HR: hazard ratio; CI: confidence interval; *Adjusted for all covariates; **Adjusted for age, number of births, material deprivation; ***Adjusted for education, social deprivation, and age.

## Discussion

### Findings of the present study and summary of literature

In this study, we assessed the association between NO_2_ and incident post-menopausal breast cancer. The adjusted HR for baseline NO_2_ was 1.10 (95% CI = 0.90, 1.34) for every 10-ppb increase in NO_2_. In our assessment of the functional form of NO_2_, we found that the highest quartile did not show an increased risk which may suggest a non-linear relationship, or there may be other factors that influence risk at higher exposure. However, when a restricted cubic spline model was fitted, we found no significant improvement in the model fit compared to the linear model. This suggests that the data does not support a nonlinear relationship between breast cancer risk and NO_2_ exposure. A significant association between increased NO_2_ and breast cancer risk has been reported in several studies, mostly nested case-control studies, or cross-sectional analyses. While some studies have reported positive and significant associations^15,20,23–25^, others have found positive but non-significant associations, consistent with our results^17,19,26,27^. Our findings are in line with two other studies. A Canadian case-control study found a positive but non-significant association between NO_2_ exposure and incident breast cancer among post-menopausal women, with an odds ratio (OR) of 1.07 (95% CI: 0.86, 1.32) for every 10-ppb increase^26^. Similarly, a study that assessed long-term exposure to air pollution and breast cancer risk among European cohorts, with 12 years of follow-up and 3,600 women developing breast cancer from the total of 75,000 post-menopausal women included in the study population, found a positive but non-significant association^19^. The HR was 1.02 per 10 µg/m3 increase (95% CI: 0.98, 1.07)^19^. The magnitude of effect in these two studies was similar to the present findings.

A US-based cohort study using LUR modeling, a method that estimates air pollution levels based on geographic and environmental data such as traffic density and land use, assessed NO_2_ exposure and observed positive associations with breast cancer risk, though these associations were not significant for all participants. However, in stratified analysis by distance to major roads, a positive and significant association was found for participants (*n* = 17,075) who lived within 500 m of major roads (combination of < 200 m and 200-<500 m categories) with an HR of 1.26 [95% CI: 1.0–1.59]^24^. In addition, a Canadian case-control study in Montreal found a significant association between air pollution and incident breast cancer, with an OR of 1.31 (95% CI: 1.00, 1.71) per 5 ppb increase in NO_2_^15^. Their study population consisted of 383 incident invasive breast cancer cases and 416 controls^15^. It is important to note the difference in sample size and exposure levels of TRAP between our study and other studies where significant association between TRAP and breast cancer risk have been reported. In our study, the mean TRAP level at baseline was 10.1 ppb. The US-based study that identified significant associations between NO_x_ and NO_2_ and breast cancer risk, reported an average NO_x_ level reported was 70 ppb in 1993, 57 ppb in 2003, and 35 ppb in 2010^24^. The absence of statistically significant associations in this study may be attributed to very low TRAP levels in Alberta^28^.

The study findings underscore the importance of considering TRAP, specifically NO_2_, in breast cancer risk management. Given the consistent positive associations observed, policymakers should recognize the potential role of NO_2_ on breast cancer incidence, while acknowledging the limitations of the current findings. Incorporating such evidence into policy considerations may involve revisiting air quality standards and urban planning regulations to mitigate NO_2_ exposure. Mechanistic studies exploring the biological pathways linking NO_2_ exposure to breast cancer development could further inform targeted policies and interventions. While the current study primarily reported no significant association, the implications for public health are substantial when considering evidence from other studies^15,19,20,24,26^,warranting a proactive approach to reducing NO_2_ exposure to potentially decrease breast cancer risk. Policymakers should consider these findings as part of a broader strategy aimed at creating healthier environments and minimizing potential environmental contributors to breast cancer incidence.

### Strengths and limitations

This study had several strengths and limitations. Unlike many previous studies where the association between TRAP and risk of breast cancer were investigated via case-control or cross-sectional studies, our study benefited from a longitudinal cohort study design. This design enabled the identification of incident breast cancer over an extended period which is more likely to capture the latency period of cancer. Furthermore, a key asset of our study was the integration of ATP data with environmental exposure information, precisely linked to each participant’s residential postal code at baseline. Moreover, the incident cancer cases were linked to the Alberta Cancer Registry, ensuring the accuracy and validity of case measurements.

In this study, we were unable to comprehensively assess participants’ total environmental exposure because NO_2_ was only measured at participants’ residence at baseline. Exposure at workplaces, schools, or places of recreation where participants may potentially spend a significant portion of time, could not be accounted for. It is possible that a participant lives in an area with low TRAP but spends time in an environment with high TRAP, and vice versa. Unfortunately, these dynamics could not be accounted for and could potentially introduce bias into our findings. However, this exposure misclassification is likely to be non-differential, which can lead to a bias towards the null, attenuating our results. Another potential limitation was our inability to accurately classify menopausal status of participants. The questionnaire that included menopause status was only administered once at the baseline and was not reassessed during the follow-up period. However, any misclassification related to menopausal status is also likely to be non-differential and may bias our results towards the null hypothesis. The potential impact of missing data, especially for important risk factors like maternal history of breast cancer, may introduce bias and limit the interpretation of our results. In addition, the small sample size further restricts the statistical power of the study, which may introduce bias and limit our ability to detect significant associations. Another limitation of this study was the lack of data on tumor characteristics, such as hormone receptor status (ER/PR).

## Conclusion

The magnitude and direction of association for NO_2_ and breast cancer risk in our study was similar to findings established from other studies, although our results were not statistically significant. Considering the wide variation in findings from the current literature, it is premature to definitively conclude a causal association between NO_2_ and breast cancer. Our result necessitate replication in larger populations, particularly populations from diverse climate conditions and variations in air pollution levels, with a specific focus on levels similar to those observed in Alberta.

## Electronic supplementary material

Below is the link to the electronic supplementary material.

Supplementary Material 1

## Data Availability

The data analyzed in this study were provided by Alberta’s Tomorrow Project. Access to individual-level data is available in accordance with the Health Information Act of Alberta and the Alberta’s Tomorrow Project (ATP) Access Guidelines and Procedures. More information can be obtained via https://myatpresearch.ca. Researchers interested in accessing the ATP data should contact ATP at the following email address: ATP.Research@albertahealthservices.ca.
