# Supplementary material for: Association between nitrogen dioxide and incident breast cancer in Alberta’s tomorrow project
Source: Sci Rep. 2025 May 30;15:19077. doi: 10.1038/s41598-025-04373-x (PMC12125178; doi:10.1038/s41598-025-04373-x)
Supplement: Supplementary file 1 — Supplementary Material 1 [file 41598_2025_4373_MOESM1_ESM.docx]

**Supplementary**

**
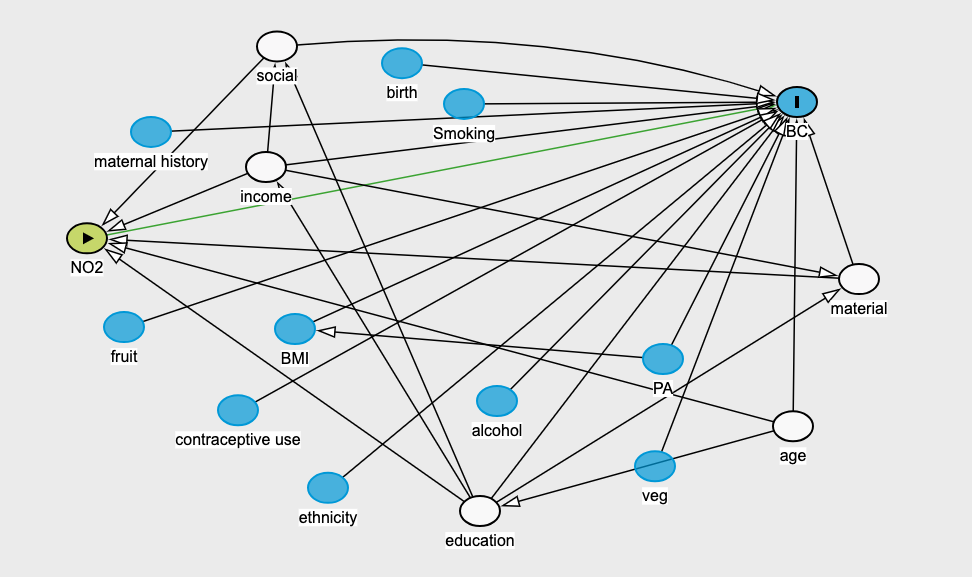
**

*Figure S1: Direct acyclic graph illustrating the relationship between breast cancer and Nitrogen Dioxide (NO_2_). Minimally sufficient adjustment set include education, social deprivation, and age.*

*
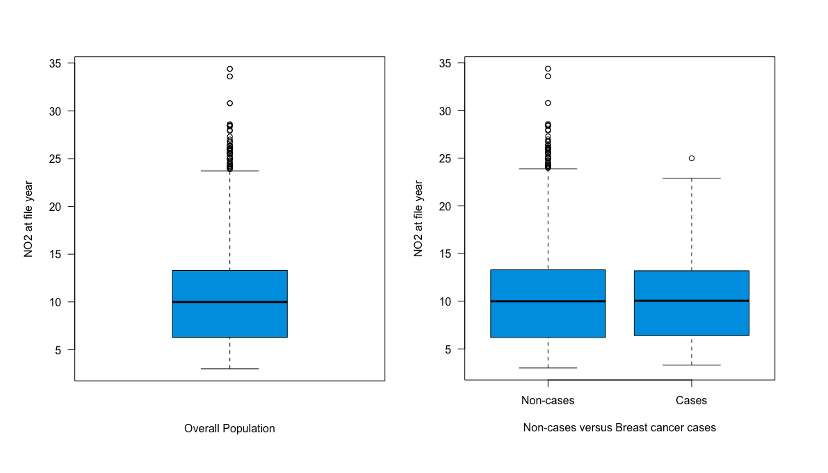
*

*Figure S2: Distribution of baseline air pollution (ppb) for breast cancer cases and non-cases.*
